# Supplementary material for: Enhanced Tailored MicroRNA Sponge Activity of RNA Pol II-Transcribed TuD Hairpins Relative to Ectopically Expressed ciRS7-Derived circRNAs
Source: Mol Ther Nucleic Acids. 2018 Sep 21;13:365–75. doi: 10.1016/j.omtn.2018.09.009 (PMC6198105; doi:10.1016/j.omtn.2018.09.009)
Supplement: Document S1. Figures S1–S4 and Tables S1–S3 [file mmc1.pdf]

OMTN, Volume *13*

Supplemental Information

Enhanced Tailored MicroRNA Sponge Activity of RNA Pol II-Transcribed TuD Hairpins Relative to Ectopically Expressed ciRS7-Derived circRNAs

Anne Kruse Hollensen, Sofie Andersen, Karina Hjorth, Rasmus O. Bak, Thomas B. Hansen, Jørgen Kjems, Lars Aagaard, Christian Kroun Damgaard, and Jacob Giehm Mikkelsen

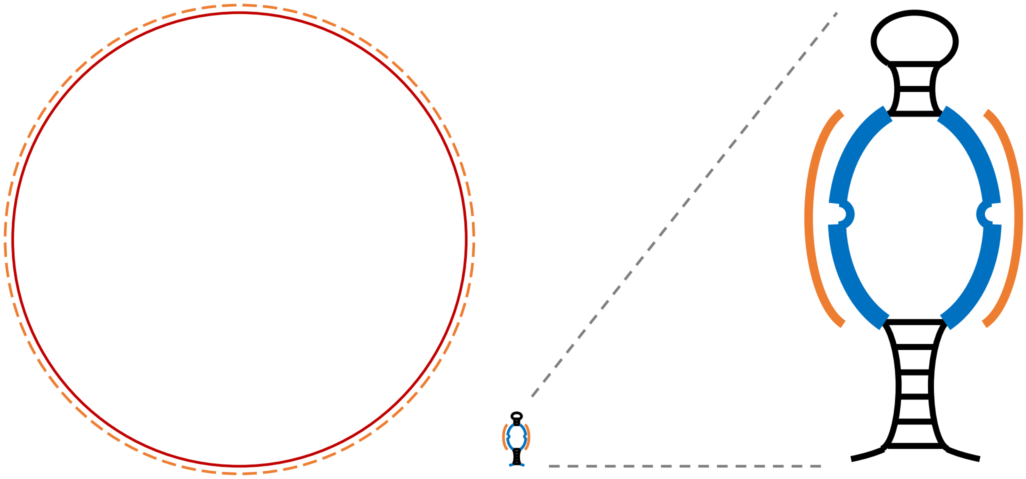

Supplementary figure S1. Schematic representation of ciRS-7 and a TuD hairpin. Processed miR-7 molecules binding to the sponges are shown in orange.

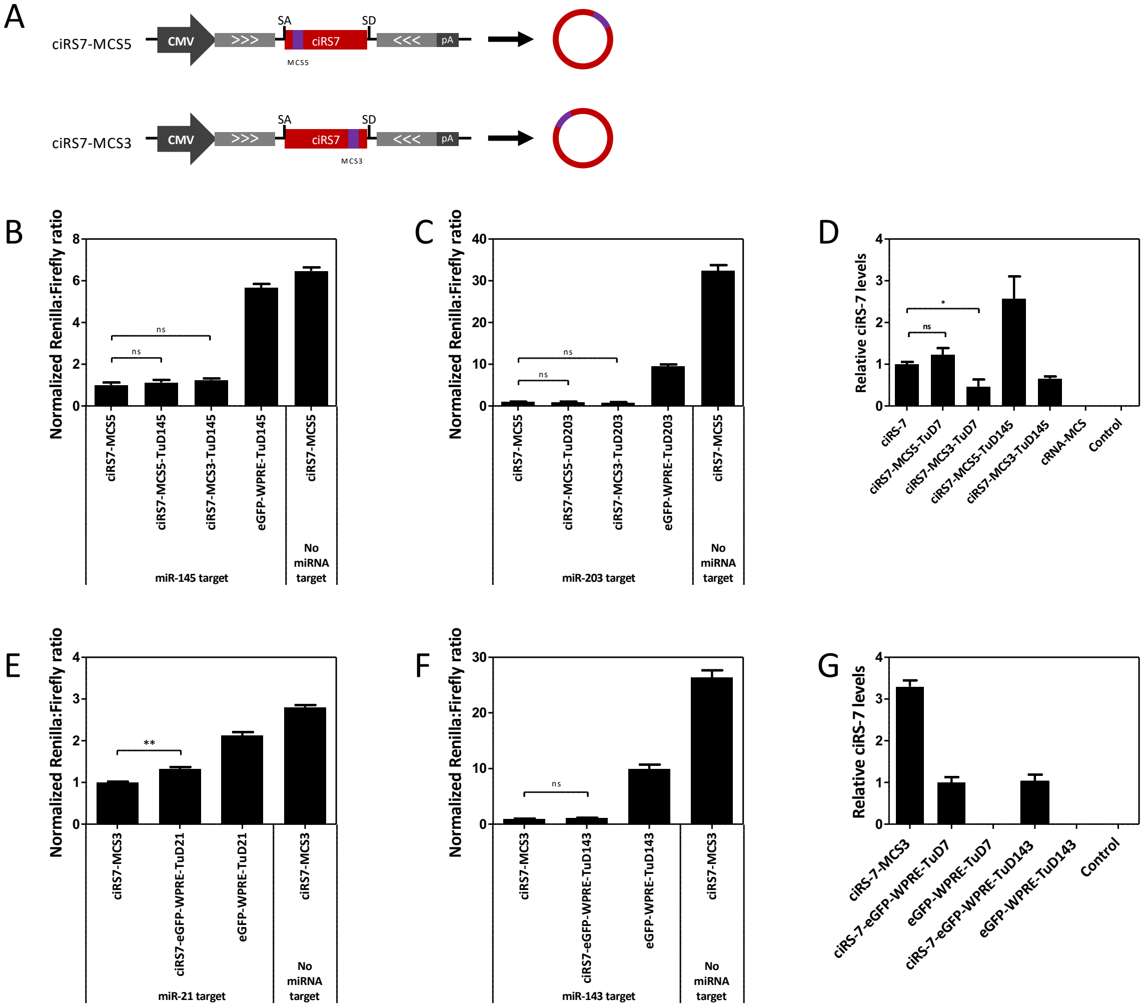

Supplementary figure S2. (A) Schematic representation of ciRS-7 expression plasmids containing multiple cloning sites in either the 5'- (MCS5) or 3'-end (MCS3) of ciRS-7. Dual-Glo luciferase assays evaluating miRNA suppression mediated by ciRS-7 carrying either TuDs targeting miR-145 (B) and -203 (C) or the eGFP-WPRE-TuD expression cassette encoding TuDs targeting miR-21 (E) and -143 (F). (D) and (G) Verification of circle formation of ciRS-7 and ciRS-7 carrying TuDs by TaqMan qPCR using primers and probe spanning the splice junction. (*) P < 0.05, (**) P < 0.01, (ns) not significant.

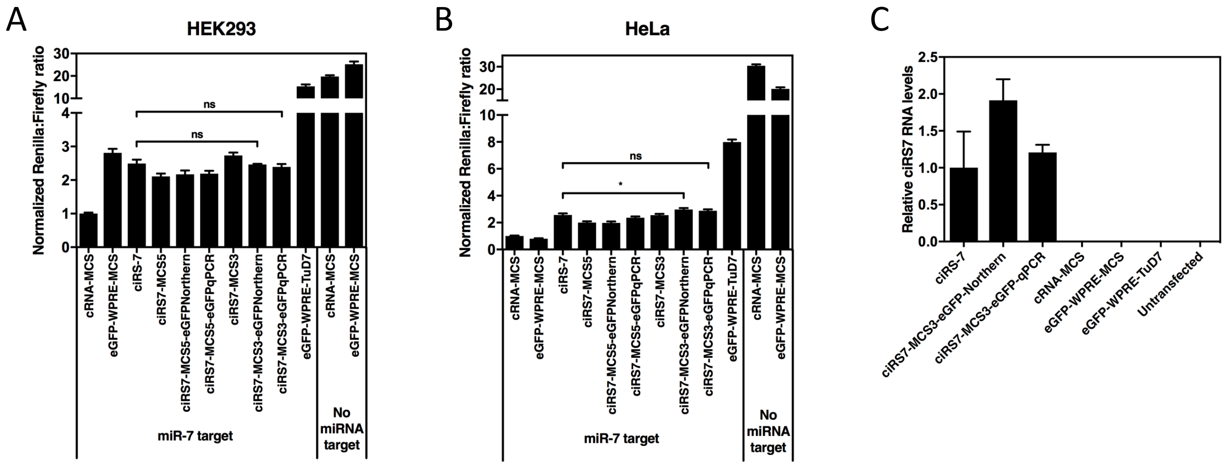

Supplementary figure S3. Dual-Glo luciferase assay in HEK293 (A) and HeLa (B) evaluating the miR-7 suppression mediated by ciRS-7 and ciRS-7 with primer and probe binding sites for either eGFP specific TaqMan qPCR (eGFP-qPCR) or Northern blot (eGFP-Northern) inserted in either ciRS7-MCS5 or ciRS7-MCS3. Both cell lines were transfected with equal molar amounts of plasmids. (C) ciRS-7 specific TaqMan qPCR using primers and probe spanning the splice junction. The same RNA samples were also used for the eGFP specific TaqMan qPCR shown in figure 3B and Northern blot shown in figure 3C. (*) $P < 0.05$, (ns) not significant.

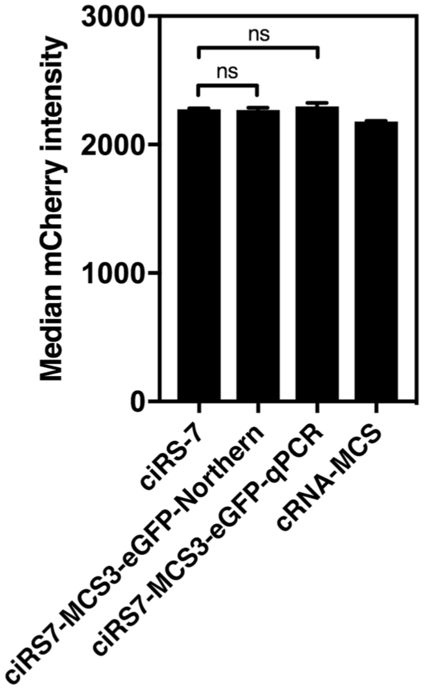

Supplementary figure S4. miR-7 suppression potential evaluated in HEK Flp-In T-rex miR7-4xmiR7-target cells with transiently expressed ciRS-7, ciRS-7-MCS3-eGFP-Northern, or ciRS-7-MCS3-eGFP-qPCR. The transfections were made using equal molar amounts of plasmids. (ns) not significant.

SUPPLEMENTARY TABLE S1: Primers

**pcDNA3/ciRS7-MCS5**	
**5141**	5’ - ccg ggg tac caa act cga gaa agg cgc gcc t - 3’
**5142**	5’ - ccg gag gcg cgc ctt tct cga gtt tgg tac c - 3’
**pcDNA3/ciRS7-MCS3**	
**5143**	5’ - ggt acc aaa ctc gag aaa ggc gcg ccg tct tcc atc aac tgg ctc aca a - 3’
**5144**	5’ - tga gcc agt tga tgg aag acg gcg cgc ctt tct cga gtt tgg tac ctt g - 3’
**pcDNA3/ciRS7-MCS5-TuD and pcDNA3/ciRS7-MCS3-TuD**	
**282**	5’ - aaa agg tac cgt atg aga cca ccc tag ccc tag ccc - 3’
**283**	5’ - aaa agg tac cca gag aga ccc agt aca agc - 3’
**pcDNA3/ciRS7-MCS5-WPRE and pcDNA3/ciRS7-MCS3-WPRE**	
**5175**	5’ - aaa ggt acc taa tca acc tct gga tta ca - 3’
**5176**	5’ - aaa ggc gcg ccg cgg gga ggc ggc cca aag g - 3’
**pcDNA3/ciRS7-eGFP-WPRE-TuD**	
**5137**	5’ - aaa gcg gcc gct ccg gaa tgg tga gca agg gcg agg ag - 3’
**5154**	5’ - aaa ggc gcg cct taa tta agc ggc cgc acc g - 3’
**pcDNA3/ciRS7-MCS5-eGFP-qPCR and pcDNA3/ciRS7-MCS3-eGFP-qPCR**	
**5244**	5’ - aaa ggt acc ctg ctg ccc gac aac cac - 3’
**5245**	5’ - aaa ggc gcg cct gtg atc gcg ctt ctc gtt - 3’
**pcDNA3/ciRS7-MCS5-eGFP-Northern and pcDNA3/ciRS7-MCS3-eGFP-Northern**	
**5230**	5’ - aaa ggt acc cta cgg cgt gca gtg ctt cag - 3’
**5231**	5’ - aaa ggc gcg cct tga aga agt cgt gct gct tc - 3’
**pcDNA3/CMV-eGFP-WPRE-TuD and pcDNA3/CMV-eGFP-WPRE-MCS**	
**5228**	5’ - aaa aag ctt gcc acc atg gtg agc aag ggc gag gag c - 3’
**5229**	5’ - aaa ggg ccc tta agc ggc cgc acc ggt ac - 3’

SUPPLEMENTARY TABLE S2: Primers and probes for TaqMan qPCR

**ciRS-7**	
**Forward primer**	5’ - gtc ttc cat caa ctg gct ca - 3’
**Reverse primer**	5’ - aac ttg aca cag gtg cca tc - 3’
**Probe**	5’ - Fam - ctt cca acg tct cca gtg tgc tga - BHQ1 - 3’
**eGFP**	
**Forward primer**	5’ - ctg ctg ccc gac aac cac - 3’
**Reverse primer**	5’ - tgt gat cgc gct tct cgt t - 3’
**Probe**	5’ - Fam - acc tga gca ccc agt ccg cc t - Tamra - 3’
**WPRE**	
**Forward primer**	5’ – ggc act gac aat tcc gtg gt - 3’
**Reverse primer**	5’ – agg gac gta gca gaa gga cg - 3’
**Probe**	5’ - Fam – acg tcc ttt cca tgg ctg ctc gc - BHQ1 - 3’
**RPLP0**	
**Forward primer**	5’ - ggc gac ctg gaa gtc caa ct - 3’
**Reverse primer**	5’ - cca tca gca cca cag cct tc - 3’
**Probe**	5’ - Fam - atc tgc tgc atc tgc ttg gag ccc a - Tamra - 3’

SUPPLEMENTARY TABLE S3: Probes for Northern blot

**eGFP**	5’ - ttg aag aag tcg tgc tgc ttc atg tgg tcg ggg tag cgg ctg aag cac tgc acg ccg tag - 3’
**B-actin**	5’ - gcc aga ttt tct cca tgt cgt ccc agt tgg tga cga tgc cgt gct cga tg - 3’
**ciRS-7**	5’ - ttg gaa gac ttg aag tcg ctg gaa gac ccg gag ttg ttg gaa gac ctt gac aca ggt gcc - 3’
**rps5**	5’ - gca gac agg ttt att ggg cag cag ctg gga aaa tca gcg gtt gga ctt ggc cac acg ctc - 3’
