# Supplementary material for: Enhanced Tailored MicroRNA Sponge Activity of RNA Pol II-Transcribed TuD Hairpins Relative to Ectopically Expressed ciRS7-Derived circRNAs
Source: Mol Ther Nucleic Acids. 2018 Sep 21;13:365–75. doi: 10.1016/j.omtn.2018.09.009 (PMC6198105; doi:10.1016/j.omtn.2018.09.009)
Supplement: Document S2. Article plus Supplemental Information [file mmc2.pdf]

Molecular Therapy
Nucleic Acids
Original Article

Enhanced Tailored MicroRNA Sponge Activity of RNA Pol II-Transcribed TuD Hairpins Relative to Ectopically Expressed ciRS7-Derived circRNAs

Anne Kruse Hollensen,[1,2] Sofie Andersen,[1] Karina Hjorth,[2] Rasmus O. Bak,[1,3] Thomas B. Hansen,[2] Jørgen Kjems,[2] Lars Aagaard,[1] Christian Kroun Damgaard,[2] and Jacob Giehm Mikkelsen[1]

[1]Department of Biomedicine, Health, Aarhus University, 8000 Aarhus C, Denmark; [2]Department of Molecular Biology and Genetics, Science and Technology, Aarhus University, 8000 Aarhus C, Denmark; [3]Aarhus Institute of Advanced Studies (AIAS), Aarhus University, 8000 Aarhus C, Denmark

As key regulators of gene expression, microRNAs (miRNAs) have emerged as targets in basic experimentation and therapy. Administration of DNA-encoded RNA molecules, targeting miRNAs through base pairing, is one viable strategy for inhibiting specific miRNAs. A naturally occurring circular RNA (circRNA), ciRS-7, serving as a miRNA-7 (miR-7) sponge was recently identified. This has sparked tremendous interest in adapting circRNAs for suppressing miRNA function. In parallel, we and others have demonstrated efficacy of expressed anti-miRNA Tough Decoy (TuD) hairpins. To compare properties of such inhibitors, we express ciRS-7 and TuD-containing miRNA suppressor transcripts from identical vector formats adapted from RNA polymerase II-directed expression plasmids previously used for production of ciRS-7. In general, markedly higher levels of miR-7 suppression with TuD transcripts relative to ciRS-7 are observed, leading to superior miRNA sponge effects using expressed TuD hairpins. Notably however, we find that individual ciRS-7 transcripts are more potent inhibitors of miR-7 activity than individual TuD7-containing transcripts, although each miR-7 seed match target site in ciRS-7 is, on average, less potent than the perfectly matched target sites in the TuD motif. All together, our studies call for improved means of designing and producing circRNAs for customized miRNA targeting to match TuD hairpins for tailored miRNA suppression.

INTRODUCTION

MicroRNAs (miRNAs) regulate gene expression at the posttranscriptional level and have emerged as key regulators of almost all biological processes. Erroneous miRNA expression may influence cell homeostasis and cause disease, and miRNAs are considered new potential drug targets.[1,2] Suppression of miRNA activity can be achieved by administration of synthetic miRNA inhibitors, including potent inhibitors based on locked nucleic acid (LNA) chemistry.[3] However, for many experimental purposes, including *in vivo* studies of miRNA function and clinical applications that may benefit from prolonged expression of miRNA inhibitors, it is attractive to establish means of expressing miRNA suppressor molecules from DNA vector systems—viral or non-viral—delivered to a certain cell type or tissue. During the last decade, a wealth of different miRNA suppression strategies based on expression of vector-encoded miRNA inhibitors has been explored. One of the most frequently used types of inhibitor, often referred to as an antagomiR, is also the structurally most simple miRNA inhibitor, consisting of a short RNA fully complementary to the target miRNA. Although the vector-encoded antagomiRs successfully have been used to suppress the activity of specific miRNAs both *in vitro* and *in vivo*,[4,5] the need for more effective miRNA inhibitors has inspired various new designs of vector-encoded miRNA inhibitors.[6–10] One of these, designated the tough decoy (TuD) miRNA sponge, has been shown to be particularly efficient.[8,11] The TuD RNA molecule is approximately 120 nt long and is designed to form a hairpin-shaped structure with stabilizing stems in both top and bottom and an intervening unpaired region consisting of two perfect miRNA target sites (Figure S1).[8] To avoid cleavage of the TuD molecule by Ago-2 upon miRNA binding, each of the miRNA target sites contains a 4-nt long bulge opposite to nucleotide 10–11 of the bound miRNA. However, the fate of the miRNAs after binding to TuD is still not clear,[8,11,12] although a high-throughput sequencing study indicated that miRNAs bound by TuDs are degraded by a tailing and trimming pathway.[13] Originally, Haraguchi and coworkers[8] expressed the TuDs as short RNAs from an RNA polymerase III (RNA pol III) promoter. However, we have previously shown equally high efficiency of RNA pol II-transcribed TuDs fused to a reporter gene and the woodchuck hepatitis virus post-transcriptional regulatory element (WPRE).[11,14] Whereas TuDs are typically exploited for suppression of a specific miRNA species and potentially families of miRNAs, sophisticated new designs allow simultaneous suppression of multiple miRNAs by clustered TuDs[15] and alleviation of small hairpin RNA off-target effects using TuDs targeting the passenger strand.[16,17]

Received 13 June 2018; accepted 17 September 2018;
https://doi.org/10.1016/j.omtn.2018.09.009.

Correspondence: Jacob Giehm Mikkelsen, Department of Biomedicine, Health, Aarhus University, 8000 Aarhus C, Denmark.
E-mail: giehm@biomed.au.dk

This is an open access article under the CC BY license (http://creativecommons.org/licenses/by/4.0/).

A few years ago, a miRNA sponge function of an endogenously expressed circular RNA (circRNA), referred to as circRNA sponge for miR-7 (ciRS-7), was described in back-to-back reports.[18,19] ciRS-7 is generated by a still not fully deduced non-canonical splicing mechanism covalently joining the 5′ and 3′ ends of an antisense transcript transcribed from the Cerebellar Degeneration-Related protein 1 (CDR1) locus and therefore is often referred to as Cdr1as. The approximately 1,500-nt-long circRNA molecule carries more than 70 seed matches for miR-7, which makes ciRS-7 capable of sponging miR-7 (Figure S1).[18] ciRS-7 is expressed in most tissues[19,20] and is potentially active in various cancer-related cellular pathways.[21] However, the expression is especially high in the brain, indicating that ciRS-7, likely among other functions, plays a role in maintenance of normal brain function.[18–20] Notably, a recent study has associated loss of ciRS-7 in the brain of mice with neuropsychiatric-like alterations.[22] Encoded by a plasmid and expressed from an RNA pol II promoter, ciRS-7 has been explored both in *in vitro* and *in vivo* studies focusing on the role of miR-7 for different pathogenic conditions.[19,23,24] Compared to conventionally used miRNA sponges expressed as linear RNAs, ciRS-7 has an advantage by being resistant to degradation by exonucleases owing to the circular structure.[18] Thus, ciRS-7 may potentially confer long-lasting suppression of miRNA activity relative to less stable types of miRNA sponges.

Vector designs exploiting formation of circular miRNA sponges by back-splicing are potentially useful for inhibiting miRNA function. In a review, written in the wake of the original ciRS-7 papers, we launched the idea that a ciRS7-based vector platform would be feasible for potent miRNA suppression based on expression of circular miRNA sponges carrying TuD motifs.[25] In the present study, we set out to investigate whether a vectorized miRNA suppression strategy based on ciRS-7 compares in capacity with that of RNA pol II-transcribed TuDs. By expressing miR7-targeting TuD and ciRS-7 sponges from plasmid backbones that were previously used for successful production of circRNAs, we show superior miRNA suppression capacity by expressing TuDs targeting miR-7 compared to ciRS-7 and only a minor improvement of the miR-7 sponge activity by inserting a TuD-7 motif in the sequence of ciRS-7. Notably, we observe that individual ciRS-7 transcripts are more potent inhibitors of miR-7 activity than individual TuD-7 transcripts, although each separate miR-7 target site in ciRS-7 is a less potent target site than the perfectly matched targets sites in the TuD hairpin. Thus, superior miRNA sponge activity of capped and polyadenylated linear RNAs carrying a TuD motif relative to circRNAs is explained primarily by marked differences in the cellular levels of the respective inhibitor RNAs.

RESULTS

miRNA-7 Suppression by miR7-Targeting TuD and ciRS-7

To compare the miRNA suppression potential of a miR7-specific TuD platform (TuD-7) with a circular ciRS-7 miRNA sponge, we constructed expression plasmids based on the ciRS-7 expression plasmid previously developed by Hansen and coworkers[18] for production of back-spliced circRNAs. Hence, TuD7-containing transcripts and ciRS-7 RNA were expressed from identical plasmid backbones (pcDNA3) driven by the CMV RNA pol II promoter (Figure 1A). In accordance with our previous work, the TuD-7 motif was fused to the 3′ end of a WPRE-containing eGFP reporter gene (eGFP-WPRE-TuD7),[11,14] whereas ciRS-7 was generated by back-splicing of a transcript containing the ciRS-7 region flanked by splice sites and regions known to be required for circle formation.

To monitor the miRNA suppression potential of the two sponging strategies, HeLa and HEK293 cells were cotransfected with a cocktail of three plasmids consisting of one of the two expression plasmids, plasmid DNA expressing miR-7, and psiCHECK reporter plasmid. The psiCHECK reporter plasmid encodes a Renilla Luciferase (RLuc) reporter gene carrying a perfect miR-7 target site in the 3′ UTR (Figure 1B), rendering RLuc protein expression regulated by binding of miR-7 to the RLuc-encoding mRNA. In addition, the psiCHECK reporter plasmid encodes a Firefly Luciferase (FLuc) reporter gene used as a control for potential variations in transfection efficiencies. As a control for miR-7 inhibition mediated by ciRS-7, a vector encoding a 62-bp multiple cloning site (MCS) between the splice acceptor (SA) and splice donor sites instead of ciRS-7 was included (cRNA-MCS), whereas an eGFP-WPRE-MCS expression cassette devoid of the TuD motifs was used as control for eGFP-WPRE-TuD-7 (Figure 1A). Reporter assays evaluating the efficiency of the two miRNA inhibitors as normalized RLuc:FLuc ratios showed in both HeLa and HEK293 cells more efficient miR-7 suppression by eGFP-WPRE-TuD7 than by ciRS-7 (Figures 1C and 1D). In HeLa cells, 10-fold higher suppression of miR-7 activity was mediated by eGFP-WPRE-TuD7 (relative to eGFP-WPRE-MCS), whereas only 3-fold higher miR-7 suppression was seen for ciRS-7 (relative to cRNA-MCS) (Figure 1C). In HEK293 cells, eGFP-WPRE-TuD7 and ciRS-7 resulted in 6- and 2-fold higher suppression of miR-7 activity, respectively, relative to the respective controls (Figure 1D). Hence, under these experimental conditions, expression of TuD-7 suppressed the activity of miR-7 3-fold more efficiently than ciRS-7. In both HeLa and HEK293 cells, controls performed with a RLuc reporter lacking the miR-7 target site showed high levels of RLuc production verifying that expression of the reporter carrying the target site was indeed tightly regulated by miR-7.

Modest Enhancement of miR-7 Suppression Mediated by ciRS-7 Carrying TuD-7

In an attempt to exploit the ciRS-7 scaffold to establish optimized TuD-mediated miRNA suppression, we examined whether heterologous sequences could be introduced inside the ciRS-7 sequence. To produce circRNA molecules carrying (1) a TuD motif, (2) an eGFP-WPRE-TuD expression cassette, or (3) WPRE, we generated a ciRS-7 expression vector with an MCS inserted at the 5′ end of ciRS-7 139 bp downstream of the SA site (MCS5) (Figure S2A). Due to the cloning procedure, a sequence of 571 bp was removed inside ciRS-7, resulting in loss of 26 miR-7 seed matches in the circular ciRS-7 RNA molecule. In addition, we generated a construct with a MCS in the 3′ end of the ciRS-7 region 82 bp upstream of the splice donor site (MCS3) (Figure S2A). Heterologous sequences were subsequently cloned into the MCS3 and MCS5 plasmids, resulting in

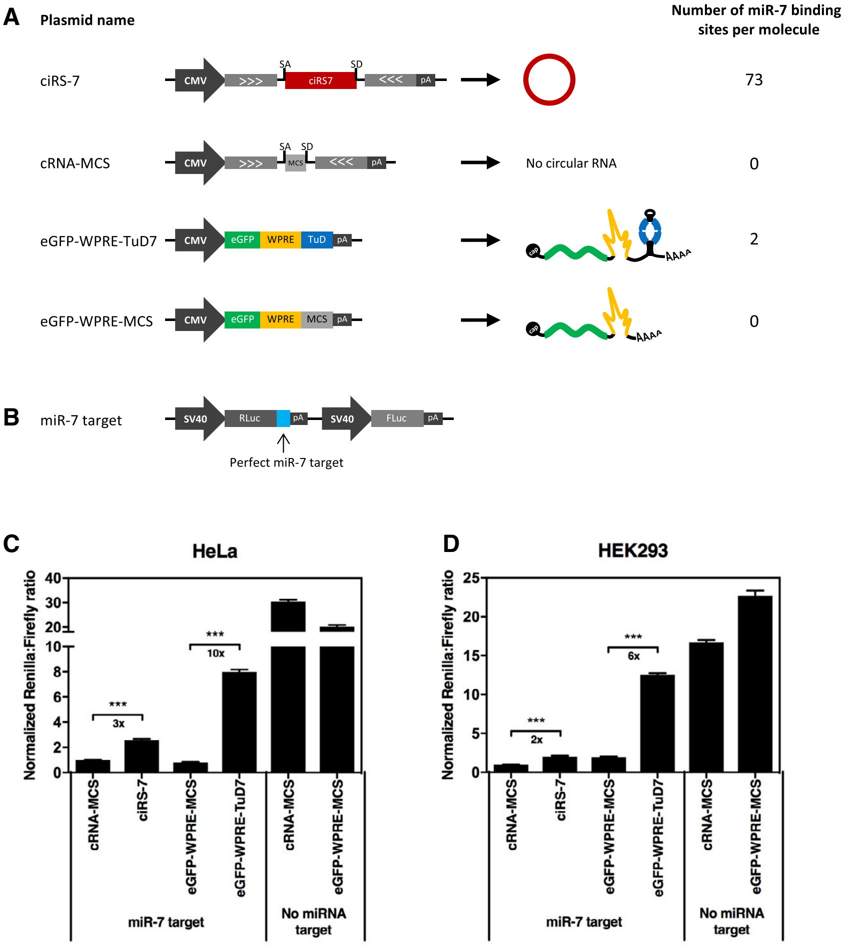

Figure 1. Superior miR-7 Suppression by miR7-Specific TuDs Compared to ciRS-7

(A) Schematic representation of ciRS-7 and TuD-7 expressed from the CMV RNA pol II promoter. SA and SD refer to splice acceptor site and splice donor site, respectively. (B) Schematic representation of the psiCHECK reporter plasmid designated miR-7 target. The miR-7 target reporter vector encodes a perfect miR-7 target site in the 3′ UTR of an RLuc reporter gene, whereas the vector designated no miRNA target encodes an RLuc reporter gene without any miRNA target sites in the 3′ UTR. Furthermore, the psiCHECK reporter plasmid encodes a FLuc reporter gene used as a control for potential variations in transfection efficiencies. Dual-Glo luciferase assays evaluating the miR-7 suppression potentials of TuD-7 and ciRS-7 in HeLa (C) and HEK293 (D) cells. The transfections were made using equal molar amounts of plasmids. Data are depicted as mean ± SEM; ***$p < 0.001$.

plasmids shown in Figures 2A–2C. Evaluation of the miRNA suppression potential by Dual-Glo luciferase assays showed a significant but only modest increase in miR-7 suppression by including the TuD hairpin in ciRS-7 (ciRS7-MCS5-TuD7 relative to the native ciRS-7 molecule) (Figure 2A). No measurable suppression of target miRNAs was observed with expression of ciRS7-MCS3-TuD7 (Figure 2A), and neither MCS5 nor MCS3 variants targeting other miRNAs (miR-145 and miR-203) showed any anti-miRNA activity (Figures S2B and S2C). We confirmed that insertion of TuDs into ciRS-7 did not hinder circRNA formation, although circRNA production seemed to be affected by the TuD insertions in a manner that was not directly predictable (Figures S2D and S2G). Overall, however, the lack of activity did not reflect reduced circle formation.

We have previously shown efficient miRNA suppression by RNA pol II-transcribed eGFP-WPRE-TuD expression cassettes encoding TuDs targeting various miRNAs.[11,14] However, Dual-Glo luciferase assays did not show any miRNA suppression activity of eGFP-WPRE-TuD7 (Figure 2B) and eGFP-WPRE-TuD-143 (Figure S2F) cassettes, targeting miR-7 and miR-143, respectively, in a circular context. For targeting of miR-21, we did indeed register suppression but to an extent that was much lower than for the corresponding RNA Pol II-transcribed TuD (Figure S2E).

Since we have shown recently that the WPRE module is essential for potent miRNA suppression activity of RNA pol II-transcribed TuD,[14] we studied the potential of WPRE to improve miR-7 suppression by ciRS-7. However, reporter assays did not verify any positive effect on miR-7 suppression by including WPRE in ciRS-7 (Figure 2C).

Higher RNA Level Explains More Efficient miR-7 Suppression by TuD-7 Relative to ciRS-7

Although we expressed eGFP-WPRE-TuD7 and ciRS-7 from identical plasmid backbones and promoters, differences in levels of linear versus back-spliced RNA could potentially explain differences in miR-7 suppression mediated by eGFP-WPRE-TuD7 relative to ciRS-7. To test this hypothesis, we constructed ciRS-7 vectors containing complementary sequences for either an eGFP-specific probe for northern blot (eGFP-Northern) or for eGFP-specific primers and probes for TaqMan qPCR (eGFP-qPCR) inserted in either ciRS7-MCS5 or ciRS7-MCS3 (Figures 3A and S2A). These vectors allowed us to quantify eGFP-WPRE-TuD7 and ciRS-7 RNA expression levels using the same primers and probes. Since Dual-Glo luciferase assays in HeLa and HEK293 cells showed only small differences in the miR-7 suppression potential of ciRS-7 containing the eGFP-Northern and eGFP-qPCR sequences in MCS3 (Figures S3A and S3B) and a ciRS7-specific TaqMan qPCR verified circle formation of both vectors (Figure S3C), these vectors were used for evaluation of eGFP-WPRE-TuD7 and ciRS-7 RNA expression levels. An eGFP-specific TaqMan qPCR assay using RNA from HeLa cells transfected with equal molar amounts of ciRS7- and eGFP-WPRE-TuD7-encoding plasmids showed more than 40-fold higher levels of eGFP-WPRE-TuD7-encoding RNA relative to RNA, linear and circRNA combined, expressed from the ciRS7-MCS3-eGFP-qPCR construct (Figure 3B). To further support

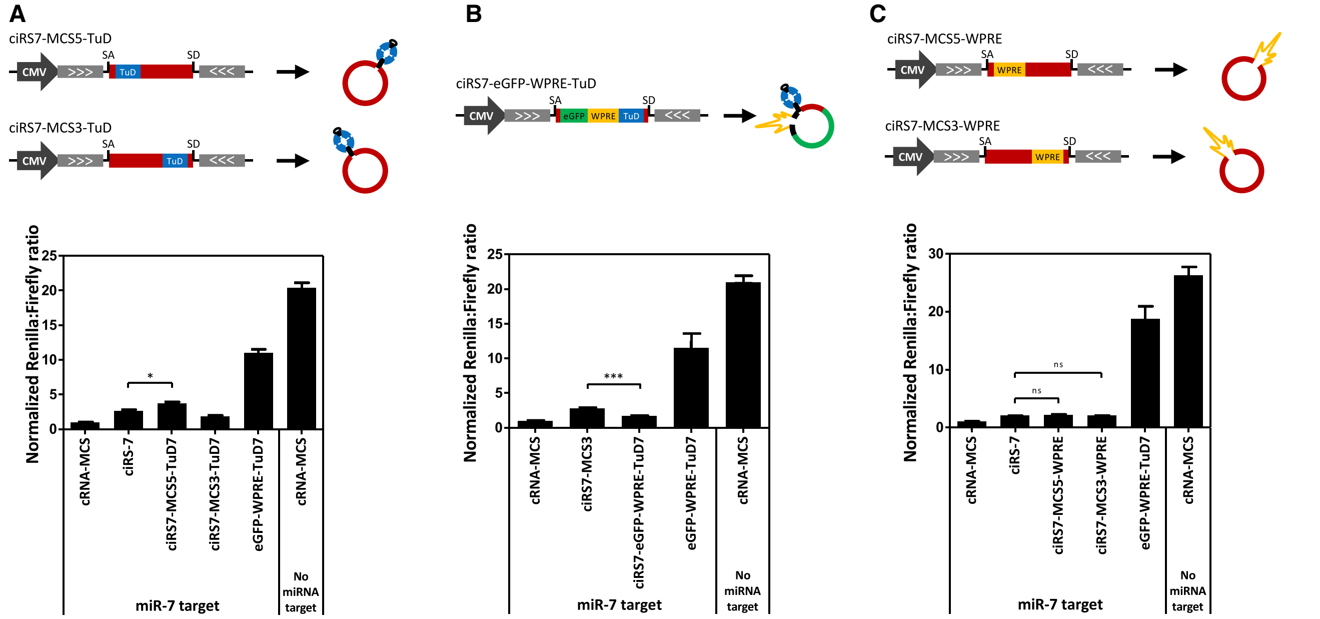

Figure 2. Modest Enhancement of miR-7 Suppression by ciRS-7 Carrying a miR7-Specific TuD

Dual-Glo luciferase assays evaluating miR-7 suppression by ciRS-7 carrying TuD-7 in either (A) MCS5 (ciRS7-MCS5-TuD) or MCS3 (ciRS7-MCS3-TuD), (B) an eGFP-WPRE-TuD-7 expression cassette (ciRS7-eGFP-WPRE-TuD), or (C) WPRE in either MCS5 (ciRS7-MCS5-WPRE) or MCS3 (ciRS7-MCS3-WPRE). Data are depicted as mean ± SEM; *p < 0.05; ***p < 0.001; ns, not significant.

the observation, we analyzed the same RNA samples by northern blotting using an eGFP-specific probe (Figure 3C). Quantification of the band intensities from the northern blot showed more than 30-fold higher levels of eGFP-WPRE-TuD7 RNA relative to ciRS7-MCS3-TuD7 (Figure 3D) and thus verified the results obtained by qRT-PCR.

Since differences in expression levels seemed to represent a plausible explanation for the superior miRNA suppression potential of TuD-7 RNA molecules compared to ciRS-7, we carried out a Dual-Glo luciferase dose-response assay to determine the minimum amount of TuD7-encoding plasmid necessary for obtaining the same level of miRNA suppression as mediated by ciRS-7. The dose-response assay showed that more than 800-fold lower amounts of eGFP-WPRE-TuD7-encoding plasmid (0.125 ng) compared to ciRS7-encoding plasmid (102 ng) still resulted in significantly higher levels of miR-7 suppression (Figure 3E). In conclusion, our findings show that the superior miRNA suppression mediated by TuD-7 compared to ciRS-7 is explained, at least partially, by marked differences in RNA expression levels.

Superior miR-7 Sponging Activity of Individual ciRS-7 Molecules Is Overshadowed by Suboptimal Expression

Due to the limited production of ciRS-7 that we reproducibly observed using the original pcDNA3/ciRS-7 plasmid,[18] we moved on to produce ciRS-7 from another plasmid, pLaccase-ciRS7, encoding ciRS-7 flanked by introns from the *Drosophila* Laccase2 gene (Figure 4A).[26]. To study the capacity of the inhibitors to suppress miR-7 in more detail, we generated a HEK Flp-In T-Rex cell line with ectopic expression of miR-7 and inducible expression of a miR7-regulated mCherry reporter gene (mCherry-4xmiR7 carrying four miR-7 seed-matching target sites) stably integrated at a single site using Flp recombination (Figure 4B). By transfections of this cell line, we confirmed improved production of ciRS-7 from the Laccase-ciRS7 construct, as measured by both northern blotting (Figures 4C and 4D) and qPCR (Figure 4E), relative to the original plasmid carrying ciRS-7 flanked by complementary intron sequences. We then transiently expressed ciRS-7, Laccase-ciRS7, and TuD-7, while simultaneously inducing expression of the mCherry reporter gene, and found that only cells expressing Laccase-ciRS7 and eGFP-WPRE-TuD7 showed levels of mCherry that were increased relative to the respective controls (Figure 4F). Although Laccase-ciRS7 was in this context a more potent miR-7 inhibitor than ciRS-7, the TuD-7 transcript was also in this cell line the most potent suppressor of miR-7.

Based on the combined expression and suppression data, we wanted to compare the miRNA suppression capacity of ciRS-7 and TuD-7 inhibitors. First, based on the capacity to target ectopically expressed miR-7 and thus induce increased mCherry expression (Figure 4F), the relative miR7-suppressive capacity of TuD-7 was 1.7 times higher than the capacity of Laccase-ciRS7 (Figure 5A). Considering the vast differences in expression levels (as determined by both qPCR and northern blotting; Figure 4), we found that the relative activity of each Laccase-ciRS7 molecule was at least 4-fold higher than the activity of eGFP-WPRE-TuD7 (Figure 5B). This is most likely explained by the high number of target sites on each ciRS7 molecule, which

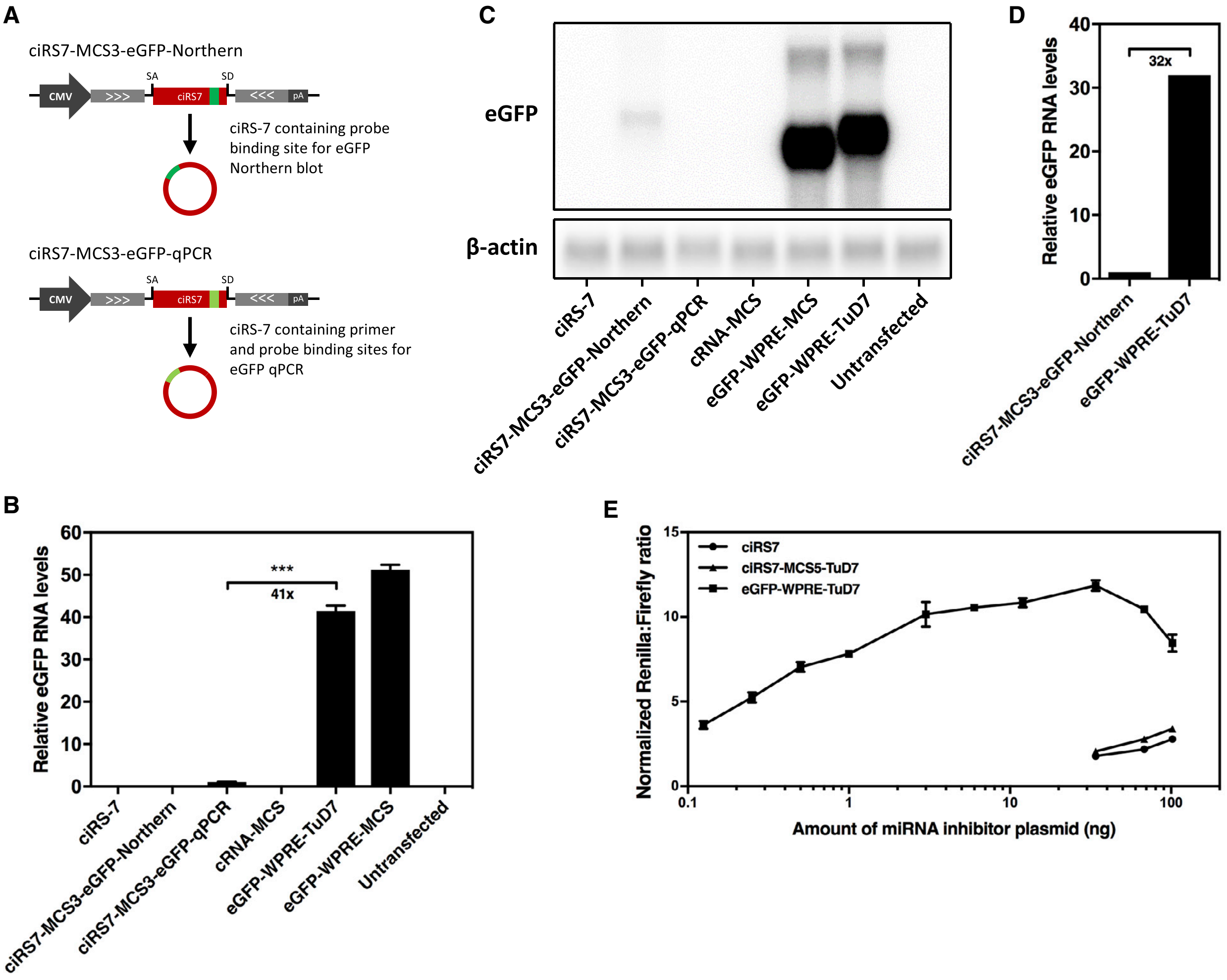

Figure 3. Higher RNA Expression Levels of TuD-7 Than ciRS-7

(A) Schematic representation of ciRS-7 encoding primer and probe binding sites for eGFP-specific TaqMan qPCR (eGFP-qPCR) or probe binding sites for northern blot (eGFP-Northern). TaqMan qPCR (B) and northern blot (C) evaluating ciRS-7 and TuD-7 expression levels in HeLa cells. (D) Quantifications of band intensities from the northern blot shown. The same RNA samples were used for the eGFP-specific TaqMan qPCR and northern blot as well as the ciRS-7 specific TaqMan qPCR shown in Figure S2C. (E) Dual-Glo luciferase assay comparing miR-7 suppression mediated by ciRS-7, ciRS7-TuD7, and TuD-7 at varying plasmid dosages. Except for the Dual-Glo luciferase assay shown in (E), the transfections were made using equal molar amounts of plasmids. (B and E) Data are depicted as mean ± SEM; ***p < 0.001.

together constitute an effective sponge despite the fact that each site is limited to a predicted miR-7 seed match. Indeed, when we then considered the sponging effect per miR-7 binding site, we found that each of the two perfect miRNA recognition sites contained in TuD-7 hairpin suppressed miR-7 activity from 3- to 9-fold (depending on the RNA quantification method) more effectively than each of the 73 miRNA targets sites in ciRS-7 and Laccase-ciRS7 (Figure 5C). We conclude that individual ciRS-7 transcripts serve as potent suppressors of miR-7 but that the overall anti-miRNA activity of TuD hairpins is higher due to overall higher expression levels and the robust sponge effect of the individual miRNA recognition sites.

DISCUSSION

Potent suppressors of miRNA function are required for studies of miRNA biology, including the identification of new miRNA targets. Moreover, discoveries of associations between various pathogenic conditions and changes in miRNA expression levels have triggered an interest for long-term safe use of miRNA sponges for genetic treatment. In clinical trials, hepatitis C infections have been treated successfully using a synthetic miRNA inhibitor targeting miR-122.[1,2] However, the relatively short lifespan and inefficient delivery to some target cells and tissues are potential obstacles for the use of synthetic suppressors of miRNA function for treatment of disease. On the contrary, vector-encoded miRNA inhibitors offer persistent

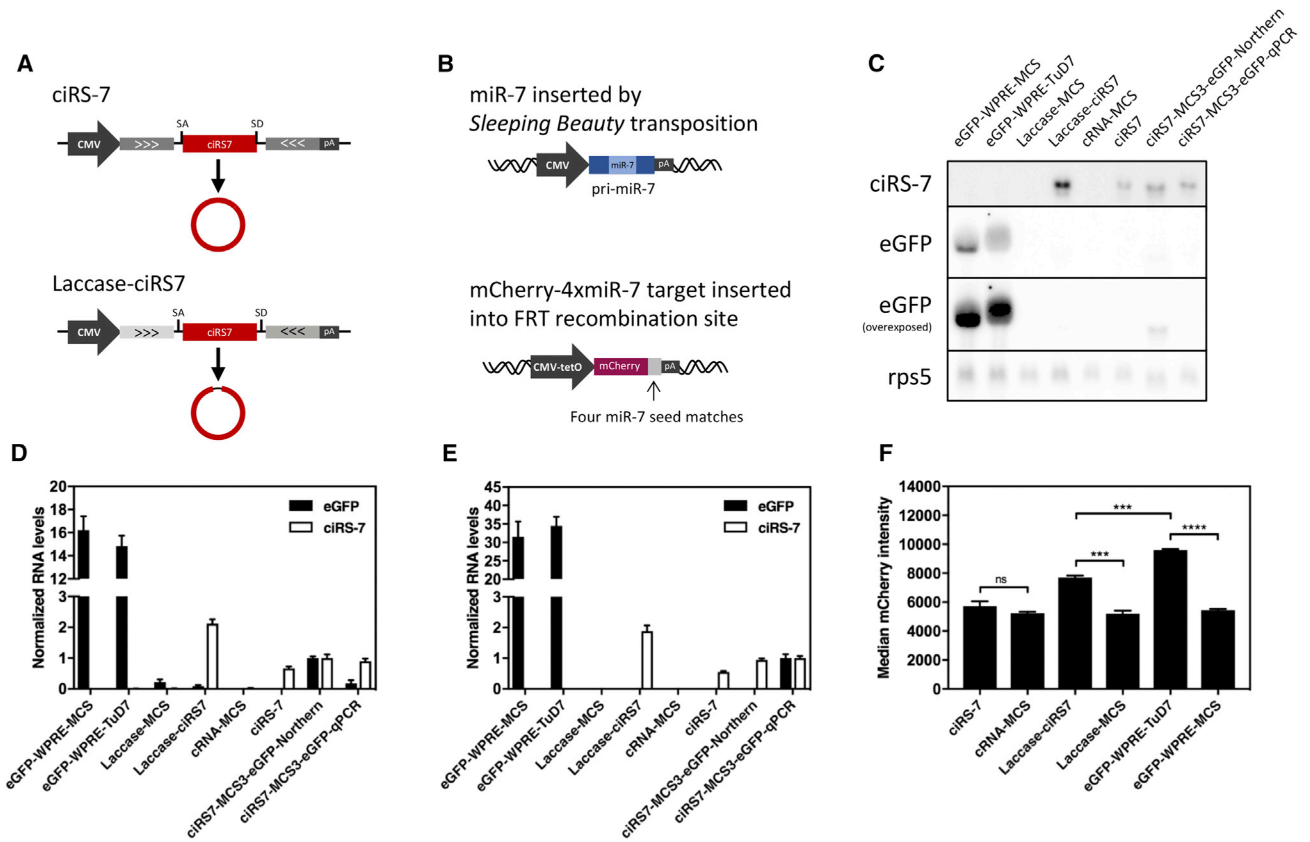

Figure 4. Superior RNA Expression Levels of TuD-7 Compared to ciRS-7

(A) Schematic representation of vectors encoding ciRS-7 flanked by complementary intron sequences derived from the endogenous sequence (ciRS-7) or introns from the *Drosophila* Laccase2 gene (Laccase-ciRS7). (B) Schematic representation of a miR-7 expression cassette stably integrated in the genome of HEK Flp-In T-Rex cells by *Sleeping Beauty* transposition (upper) and an expression cassette encoding mCherry with four canonical miR-7 binding sites in the 3′ UTR (mCherry-4xmiR7-target) inserted in the genomic FRT-site of HEK Flp-In T-Rex cells by Flp recombination (lower). Northern blot (C) and TaqMan qPCR (E) evaluating ciRS-7, Laccase-ciRS7, and TuD-7 expression levels in HEK Flp-In T-Rex miR7-4x miR7-target cells. (D) Quantifications of band intensities from the northern blot shown. For the comparison of ciRS-7, Laccase-ciRS7, and eGFP-WPRE-TuD-7 RNA levels, the expression of ciRS7-MCS3-eGFP-qPCR or ciRS7-MCS3-eGFP-Northern was used to normalize ciRS-7 and eGFP expression levels measured by qPCR or northern blot, respectively. The ciRS-7 inhibitor effect was unaffected by insertion of eGFP fragments used for quantification (Figure S4) (F) miR-7 suppression potential of ciRS-7, Laccase-ciRS7, and TuD-7 in HEK Flp-In T-Rex miR7-4xmiR7-target cells evaluated by flow cytometry. The transfections were made using equal molar amounts of plasmids. Data are depicted as mean ± SEM; ***$p < 0.001$; ****$p < 0.0001$; ns, not significant.

and potentially tissue-specific or inducible expression by use of viral or non-viral vector systems facilitating stable gene expression, for example by genomic insertion.[25]

In the present study, we showed in three different cell lines superior miRNA suppression by TuD-7 compared to ciRS-7, both inhibitors expressed by RNA pol II from transfected plasmid DNA. Notably, although we showed that the expression levels of vector-encoded ciRS-7 are generally low, recent studies have utilized the exact same expression plasmid for studies of the influence of miR-7 on brain development, insulin secretion, and myocardial infarction.[19,23,24] In addition, we investigated the idea that circRNAs equipped with a TuD motif could further improve miRNA suppression potential of TuDs. Nevertheless, only slightly increased levels of miRNA suppression were observed for some of the studied miRNAs by using ciRS-7 as a carrier of either a TuD hairpin or the longer eGFP-WPRE-TuD cassette. Although we recently have shown improved TuD-mediated miRNA suppression by fusion of RNA pol II-transcribed TuDs to WPRE,[14] WPRE did not have any beneficial effect on miR-7 suppression by ciRS-7.

As verified by both qPCR and northern blotting, the overall higher levels of miRNA suppression mediated by TuD-7 compared to ciRS-7 was a result primarily of a marked difference in expression levels (typically in the range of 30- to 40-fold) of the two types of miRNA inhibitors. Thus, although identical CMV promoters were used for RNA pol II-directed expression of TuD-7 and ciRS-7, different production levels were observed for the two miRNA inhibitors. Since the biogenesis of ciRS-7 involves additional processing including formation of a covalent junction between two splice sites

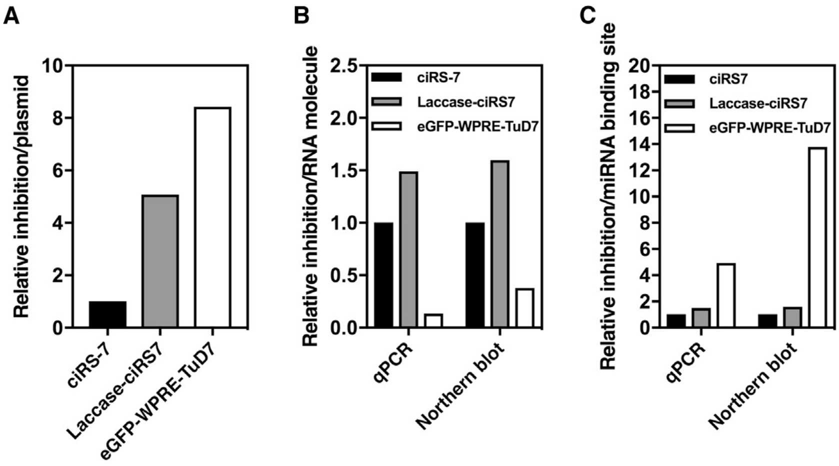

Figure 5. Relative miR-7 Inhibition Capacities of ciRS-7, Laccase-ciRS7, and TuD-7 Transcripts

(A) Relative miR-7 inhibition by ciRS-7, Laccase-ciRS7, or TuD-7 normalized to the amount of plasmid. (B) Relative miR-7 inhibition capacity the three inhibitors presented as inhibition potential per RNA molecule. (C) Relative inhibition by ciRS-7, Laccase-ciRS7, or TuD-7 presented as inhibition capacity per miRNA binding site contained in ciRS-7 (73 miR-7 binding sites), Laccase-ciRS7 (73 miR-7 binding sites), and TuD-7 (2 miR-7 binding sites). Relative inhibition capacities are based on results shown in Figures 4D–4F.

in a non-canonical fashion, this may explain the reduced number of effective ciRS-7 molecules relative to linear eGFP- and WPRE-fused TuD-7. Production of ciRS-7 was improved by expressing a ciRS-7 variant flanked by introns from the *Drosophila* Laccase2 gene.[26] In transfected HEK Flp-In T-Rex cells, increased ciRS-7 production correlated with higher levels of miR-7 suppression (Figure 4F), and when we took variations in expression levels into account, we found that the relative miR-7 suppression level per molecule was higher for ciRS-7 expressed from both the original and the improved laccase context relative to the TuD-containing transcript (Figure 5B).

Obviously, the two types of sponges exhibit other essential differences that are likely to contribute to the differences in the observed miRNA suppression potential. As opposed to the two miRNA target sites carried by TuD-7, ciRS-7 carries a total of up to 73 miRNA potential target sites for miR-7. When we considered the relative suppression capacity per miRNA target site, we noted markedly higher activity for each target site in the TuD hairpin (Figure 5C). This is not surprising, as the miR-7 target sites within ciRS-7 are only predicted seed matches that most likely establish a less-persistent interaction between the miRNA and the miRNA inhibitor, and it is not known if all potential target sites are accessible for miRNA binding. In the TuD hairpin, in contrast, both sites exhibit a near-perfect match between TuD miRNA target sites and the miRNA, with central bulges to avoid endocleavage.[8,18] Molecular and structural differences between the two types of sponges may also affect delivery using virus-based delivery systems. Although we did not move on to investigate viral vector-based delivery, the repetitive nature of ciRS-7 is likely to induce frequent recombination during reverse transcription of lentiviral vector RNA. Although size limitations may apply in adeno-associated virus (AAV)-based vectors, this vector system is more likely to reliably deliver circRNA expression cassettes.

Our studies consolidate earlier findings demonstrating that eGFP- and WPRE-fused TuD molecules are effective miRNA sponges[11,14] but also show that vector-based circRNA expression strategies need further optimization to achieve miRNA suppression activities matching the sponge effects of more potently expressed TuD hairpins. Our results point to insufficient expression of the circular inhibitor as a main explanation for reduced anti-miR7 activity of ciRS7-derived sponges relative to TuD-7 transcripts, at least in transiently transfected cell lines. However, notably, by relating sponge expression levels and miRNA suppression capacity, we also show that individual ciRS-7 transcripts are in fact more potent inhibitors of miR-7 activity than individual TuD-7 transcripts. This analysis also indicated that the separate miR-7 target sites in ciRS-7 each contributed less to the overall anti-miRNA activity than the perfectly matched miRNA targets sites in the TuD hairpin. Although it seems attractive to engineer a ciRS7-derived sponge with 70+ near-perfect miRNA target sites mimicking the sites in TuD hairpins, we deemed this approach unsuitable for an easy customizable vector delivery approach.

Despite the attractive properties of circular sponges as inhibitors that are potentially stable due to the circular conformation, vector-encoded RNA pol II-transcribed TuD are more easily customized for suppression of specific miRNAs and due to simpler means of expression recommended for potent suppression of miRNAs in standard cell assays. Although circRNAs carrying miRNA target sites may not allow easy modular assembly without additional tests of expression and circle formation capacity from each individual construct, it cannot be excluded, however, that vector-encoded ciRS-7 may have unique properties *in vivo*, e.g., in the brain, and may be attractive for use in particular tissues, in which miRNA suppression may benefit from the increased stability of circRNA. However, reduced levels of back-spliced anti-miRNA RNA molecules represent a potential challenge for the development and use of current circle generation strategies for production of potent miRNA suppressor molecules. Although first recognized as a miRNA sponge, it should be noted also that endogenous ciRS-7 may serve primarily as a source and carrier of miR-7, protecting the miRNA from degradation. This notion is supported by the finding that depletion of ciRS-7 in the mouse leads to downregulation of miR-7 at the posttranscriptional level and disturbance of normal brain function.[22] By further scrutinizing strategies for expression and processing of circRNA as well as incorporation of artificial more inhibitory miRNA target sites, one may be able to “vectorize” ciRS-7 production in a way

that facilitates both optimal vector delivery and expression. However, such tailored use of circRNAs for suppressing and potentially stabilizing miRNA species using vector delivery technologies may be challenged by the levels of expression and circle formation that may vary in an unpredictable manner dependent on the genetic content of the circle, resulting in insufficient levels of anti-miR molecules.

MATERIALS AND METHODS

Vector Construction

To allow subsequent insertion of TuDs, WPRE, and eGFP primer and probe binding sites for qPCR and northern blot in ciRS-7, the pcDNA3/ciRS7 plasmid[18] was modified to contain MCS sequences inside the ciRS-7 sequence. Oligonucleotides encoding the MCSs were phosphorylated, annealed, and inserted into either Kpn2I- or PflmI-digested pcDNA3/ciRS7 for generation of plasmids designated pcDNA3/ciRS7-MCS5 and pcDNA3/ciRS7-MCS3, respectively. For generation of ciRS-7 molecules carrying TuD-7, TuD-145, or TuD-203, TuDs were PCR-amplified from pCCL/PGK-eGFP-H1-TuD[11] and inserted into KpnI-digested pcDNA3/ciRS7-MCS5 or pcDNA3/ciRS7-MCS3. The resulting plasmids were designated pcDNA3/ciRS7-MCS5-TuD and pcDNA3/ciRS7-MCS3-TuD. Likewise, WPRE and eGFP primer and probe binding sites for qPCR and northern blot were PCR-amplified from pT2/CMV-eGFP-WPRE-MCS.SV40-neo[14] and inserted into KpnI/AscI-digested pcDNA3/ciRS7-MCS5 or pcDNA3/ciRS7-MCS3, resulting in plasmids designated pcDNA3/ciRS7-MCS5-WPRE, pcDNA3/ciRS7-MCS3-WPRE, pcDNA3/ciRS7-MCS5-eGFP-qPCR, pcDNA3/ciRS7-MCS3-eGFP-qPCR, pcDNA3/ciRS7-MCS5-eGFP-Northern, and pcDNA3/ciRS7-MCS3-eGFP-Northern. The eGFP-WPRE-TuD expression cassettes carrying TuD-7, TuD-21, or TuD-143 were PCR-amplified from pT2/CMV-eGFP-WPRE-TuD.SV40-neo[14] and inserted into Kpn2I/AscI-digested pcDNA3/ciRS7-MCS3. The resulting vectors were designated pcDNA3/ciRS7-eGFP-WPRE-TuD.

To create pcDNA3 plasmids expressing the eGFP-WPRE-TuD or eGFP-WPRE-MCS molecules from the same promoter as ciRS-7, the eGFP-WPRE-TuD and eGFP-WPRE-MCS fragments were PCR-amplified from pT2/CMV-eGFP-WPRE-TuD.SV40-neo[14] and inserted into HindIII/ApaI-digested pcDNA3/ciRS7, resulting in vectors designated pcDNA3/CMV-eGFP-WPRE-TuD and pcDNA3/CMV-eGFP-WPRE-MCS. TuDs expressed from the plasmids designated pT2/CMV-eGFP-WPRE-TuD.SV40-neo and pCCL/eGFP-TuD were made as previously described in Bak et al.[11] and Hollensen et al.[14,15]

Plasmids for expression of miR-7, miR-143, miR-145, miR-203, and psiCHECK vectors containing perfect miRNA target sites in the 3′ UTR of the RLuc reporter gene were constructed as described previously in Primo et al.[5], Bak et al.,[11], and Hollensen et al.[15] In brief, primary miRNAs (pri-miRNAs) were PCR-amplified from human genomic DNA and inserted into NotI-digested pT2/CMV-eGFP.SV40-neo,[27] resulting in a plasmid designated pT2/CMV-miR-SV40-neo. To create the psiCHECK reporter plasmids, oligonucleotides encoding perfect miRNA target sites were inserted into XhoI/NotI-digested psiCHECK-2 (Promega, Madison, WI, USA), resulting in plasmids designated psiCHECK-miR-target.

To generate plasmids for stable integration and tetracycline-inducible expression of an mCherry reporter gene with four canonical miR-7 binding sites in the 3′ UTR, pPC-mCherry-miR-7 was digested with HindIII and ApaI, and the resulting DNA fragment encoding mCherry-4xmiR7-target was inserted into HindIII-/ApaI-digested pcDNA5-FRT/TO, resulting in a plasmid designated pcDNA5-FRT/TO-mCherry-4xmiR7.

Sequences of all used primers are specified in Table S1. All plasmids were verified by sequencing (GATC Biotech, Constance, Germany).

Cell Culturing

HEK293, HeLa, and HEK Flp-In T-Rex cells were cultured at 37°C in 5% (v/v) CO_2 and maintained in DMEM (Lonza, Basel, Switzerland) supplemented with 5% fetal calf serum (Sigma-Aldrich, Milwaukee, WI, USA), penicillin (100 U/mL) (Sigma-Aldrich, Milwaukee, WI, USA), and streptomycin (0.1 mg/mL) (Sigma-Aldrich, Milwaukee, WI, USA).

Generation of Cell Lines

The *Sleeping Beauty* transposon system was used for generation of a cell line with stable overexpression of miR-7. One day before transfection, HEK Flp-In T-Rex cells were seeded at a density of 20×10^4 cells/well in 6-well plates. Transfections were carried out using 450 ng pT2/CMV-miR-7.SV40-neo and 50 ng of either a plasmid encoding an active SB transposase (pCVM-SB100X) or an inactive SB transposase (pCMV-mSB) using X-tremeGENE 9 (Roche, Basel, Switzerland) according to manufacturer's protocol. One day after transfection, the cells were reseeded in appropriate dilutions. Selection was carried out using cell culture medium containing 1,200 μg/mL G418 (Sigma). After approximately 3 weeks of selection, single colonies were isolated and expanded. miR-7 overexpression was verified by qRT-PCR. RNA was purified using TRI Reagent (Sigma-Aldrich, Milwaukee, WI, USA) according to USA). First-strand cDNA synthesis was carried out using the TaqMan MicroRNA Reverse Transcription Kit (Applied Biosystems, Foster City, CA, USA). Subsequently, qPCR reactions were prepared using the Maxima Probe qPCR Master Mix (Thermo Fisher Scientific, Massachusetts, USA) and TaqMan primers and probes specific for miR-7 or U48 (Applied Biosystems, Foster City, CA, USA). Finally, RNA levels were quantified using a LightCycler 480 (Roche, Basel, Switzerland), and miR-7 and U48 levels were calculated using the relative standard curve method. One HEK Flp-In T-Rex miR-7 clone with an intermediate level of miR-7 overexpression was selected for generation of a cell line with stable integration and tetracycline-inducible expression of an mCherry reporter gene with four canonical miR-7 binding sites in the 3′ UTR. One day before transfection, HEK Flp-In T-Rex cells were seeded at a density of 10^6 cells/dish in a 6-cm dish. The transfection was carried out using 1 μg pcDNA5-FRT/TO-4xmiR7, 9 μg Flp-recombinase expression plasmid (pOG44),

and Lipofectamine 2000 (Thermo Fisher Scientific, Massachusetts, USA) according to manufacturer's protocol. One day after transfection, cells were transferred to a T175 cell culture flask. Selection using medium supplemented with 100 μg/ml Hygromycin B (Thermo Fisher Scientific, Massachusetts, USA), and 10 μg/mL Basticidine (Thermo Fisher Scientific, Massachusetts, USA) was initiated 2 days after transfection. The resulting cell line designated HEK Flp-In T-Rex miR-7 + mCherry4xmiR7-target with stable miR-7 overexpression and inducible expression of mCherry-4xmiR7-target were hereafter kept under selection.

Dual-Glo Luciferase Assays

Dual-Glo luciferase assays were performed as previously described in Bak et al.[11] and Hollensen et al.[15]. In brief, HEK293 or HeLa cells were seeded in white 96-well plates at a density of 3,000 cells/well or 5,000 cells/well, respectively, 1 day before transfection. All cotransfections were carried out using X-tremeGENE 9 (Roche, Basel, Switzerland) according to manufacturer's protocol, 6 ng of psiCHECK reporter plasmid, and 102 ng miRNA inhibitor plasmid. However, for experiments shown in Figures 1A, 1B, 4B, S3A, and S3B, 102 ng ciRS-7 expression plasmid and equal molar amounts of the other used miRNA inhibitor plasmids were used. Due to relative low endogenous expression of miR-7, miR-143, miR-145, and miR-203,[11,14] 3 ng of miRNA expression plasmid were included for studies of suppression of these miRNAs. Two days after transfection, luciferase expression levels were measured using the Dual-Glo Luciferase Assay System (Promega, Madison, WI, USA) according to manufacturer's protocol on a multisample platereading luminometer (Berthold, Bad Wildbad, Germany). Data are presented as RLuc expression levels relative to the FLuc expression levels and normalized to a negative control.

Flow Cytometry

One day before transfection, HEK Flp-In T-Rex miR7-mCherry-4xmiR7-target cells were seeded at a density of 3×10^5 cells/well in 6-well plates or for comparison of miR-7 inhibition by ciRS-7, ciRS7-MCS3-eGFP-Northern, and ciRS7-MCS3-eGFP-qPCR 8×10^5 cells/well in 6-well plates. Transfections were carried out with 1 μg ciRS-7 expression plasmid and equal molar amounts of the other miRNA inhibitor plasmids using Lipofectamine 2000 (Thermo Fisher Scientific, Massachusetts, USA) according to manufacturer's protocol and cell culture medium supplemented with tetracycline (Thermo Fisher Scientific, Massachusetts, USA) (final concentration, 20 ng/mL). pcDNA3/PL was used as filler plasmid. Two days after transfection, cells were harvested and mCherry expression levels were analyzed on a BD LSRFortessa (BD Biosciences, Franklin Lakes, NJ, USA).

Northern Blot

For comparison of ciRS-7 and eGFP-WPRE-TuD-7 expression levels in HeLa cells, cells were seeded at a density of 1.5×10^6 cells/dish in 10-cm dishes. Transfections were carried out with 10 μg ciRS-7 expression plasmid and equal molar amounts of the other used miRNA inhibitor plasmids using 30 μL TurboFect Transfection Reagent (Thermo Fisher Scientific, Massachusetts, USA) according to manufacturer's protocol. pUC19 was used as filler plasmid. Two days after transfection, RNA was purified using TRI Reagent (Sigma-Aldrich, Milwaukee, WI, USA) according to manufacturer's protocol. 15 μg RNA (5 μg from each of three independent transfections) was separated on a 1% denaturing agarose gel and transferred to a Hybond membrane (GE Healthcare, Buckinghamshire, UK). The membrane was hybridized with eGFP- or β-actin-specific [^{32}P]-end-labeled oligonucleotides (sequences are specified in Table S3) overnight and subsequently exposed on phosphorimager screens and visualized on a Molecular Imager FX (Bio-Rad, Hercules, California, USA). Intensities of the bands were analyzed using the Quantity One software.

For comparison of ciRS-7 and eGFP-WPRE-TuD-7 expression levels in HEK Flp-In T-Rex miR7-mCherry-4xmiR7-target cells, cells were seeded at a density of 8×10^5 cells/well in 6-well plates. Transfections were carried out with 1 μg ciRS-7 expression plasmid and equal molar amounts of the other miRNA inhibitor plasmids using Lipofectamine 2000 (Thermo Fisher Scientific, Massachusetts, USA) according to manufacturer's protocol and cell culture medium supplemented with tetracycline (Thermo Fisher Scientific, Massachusetts, USA) (final concentration, 20 ng/mL). pcDNA3/PL was used as filler plasmid. Two days after transfection, RNA was purified using TRIzol (Thermo Fisher Scientific, Massachusetts, USA) according to manufacturer's protocol. 15 μg RNA was separated on a 1.2% formaldehyde-agarose gel and transferred to a Hybond membrane (GE Healthcare, Buckinghamshire, UK). The membrane was hybridized with ciRS7-, eGFP-, or rps5-specific [^{32}P]-end-labeled oligonucleotides (sequences are specified in Table S3) overnight and subsequently exposed on phosphorimager screens and visualized on a Typhoon FLA 9500 (GE Healthcare, Piscataway, NJ, USA). Intensities of the bands were analyzed using the Image Studio software (Li-Cor Biosciences, NE, USA).

qPCR

For analysis of ciRS7-MCS-TuD and ciRS7-eGFP-WPRE-TuD expression levels, 1×10^5 cells/well or 1.5×10^5 cells/well were seeded in 6-well plates, respectively. One day after seeding, the cells were transfected with 1 μg expression plasmid using either TurboFect Transfection Reagent (Thermo Fisher Scientific, Massachusetts, USA) or X-tremeGENE 9 (Roche, Basel, Switzerland) according to the manufacturer's protocols. Two days after transfection, total RNA was purified using the E.Z.N.A. Total RNA Kit I (Omega Bio-tek, Norcross, GA, USA). The RNA was treated with DNase I (Thermo Fisher Scientific, Massachusetts, USA), and first-strand cDNA synthesis was carried out using the Maxima First Strand cDNA Synthesis Kit for qPCR (Thermo Fisher Scientific, Massachusetts, USA).

For comparison of ciRS-7 and eGFP-WPRE-TuD7 expression levels, the same RNA samples as for northern blotting were used. The RNA was treated with DNase I (Thermo Fisher Scientific, Massachusetts, USA), and cDNA synthesis was carried out using Random Hexamer

Primer (Life Technologies, Carlsbad, CA, USA) and SuperScript II Reverse Transcriptase (Life Technologies, Carlsbad, CA, USA) or the Maxima First Strand cDNA Synthesis Kit for qPCR (Thermo Fisher Scientific, Massachusetts, USA).

For all experiments, mRNA expression levels were measured using the Maxima Probe qPCR Master Mix (Thermo Fisher Scientific, Massachusetts, USA) and gene-specific TaqMan primers and probes (sequences are specified in Table S2). RPLP0 was used as reference gene. A LightCycler 480 (Roche, Basel, Switzerland) or AriaMx Real-Time PCR System (Agilent Technologies, CA, California, USA) were used for quantification of mRNA levels, and the relative standard curve method was used for calculations of relative mRNA levels.

Statistical Analysis

All p values were calculated by a two-tailed Student's t test to test the null hypothesis of no difference between the two compared groups. The assumption of equal variances was tested by an F test. $p < 0.05$ was considered statistically significant. Data are presented as mean + SEM.

SUPPLEMENTAL INFORMATION

Supplemental Information includes four figures and three tables and can be found with this article online at https://doi.org/10.1016/j.omtn.2018.09.009.

AUTHOR CONTRIBUTIONS

The study was conceived and designed by A.K.H., R.O.B., L.A., C.K.D., and J.G.M. with assistance from T.B.H. and J.K. A.K.H., S.A., and K.H. performed the experiments. A.K.H. and J.G.M. wrote the manuscript and assembled the figures.

CONFLICTS OF INTEREST

T.B.H. and J.K. are listed as inventors on a patent filed by Aarhus University concerning the use of artificial circRNA as microRNA sponges. The other authors declare no conflict of interest.

ACKNOWLEDGMENTS

We would like to thank Lisbeth Dahl Schrøder for technical assistance. This work was made possible through support from the Lundbeck Foundation (R126-2012-12456), the Novo Nordisk Foundation (NNF17OC0029042), Carl og Ellen Hertz' legat til læge-og naturvidenskaben, Harboefonden, Arvid Nilssons Fond, Fonden af 1870, Direktør Emil C. Hertz og hustru Inger Hertz' Fond, and Fabrikant Ejnar Willumsens Legat. A.K.H. was enrolled at and funded by the Graduate School of Health, Aarhus University, and is currently supported by stipends granted by the Novo Nordisk Foundation (NNF15OC0017598; NNF17OC0028804) to C.K.D.

REFERENCES

1. Janssen, H.L., Reesink, H.W., Lawitz, E.J., Zeuzem, S., Rodriguez-Torres, M., Patel, K., van der Meer, A.J., Patick, A.K., Chen, A., Zhou, Y., et al. (2013). Treatment of HCV infection by targeting microRNA. N. Engl. J. Med. *368*, 1685–1694.
2. van der Ree, M.H., van der Meer, A.J., de Bruijne, J., Maan, R., van Vliet, A., Welzel, T.M., Zeuzem, S., Lawitz, E.J., Rodriguez-Torres, M., Kupcova, V., et al. (2014). Long-term safety and efficacy of microRNA-targeted therapy in chronic hepatitis C patients. Antiviral Res. *111*, 53–59.
3. Juliano, R.L. (2016). The delivery of therapeutic oligonucleotides. Nucleic Acids Res. *44*, 6518–6548.
4. Scherr, M., Venturini, L., Battmer, K., Schaller-Schoenitz, M., Schaefer, D., Dallmann, I., Ganser, A., and Eder, M. (2007). Lentivirus-mediated antagomir expression for specific inhibition of miRNA function. Nucleic Acids Res. *35*, e149.
5. Primo, M.N., Bak, R.O., Schibler, B., and Mikkelsen, J.G. (2012). Regulation of pro-inflammatory cytokines TNFα and IL24 by microRNA-203 in primary keratinocytes. Cytokine *60*, 741–748.
6. Ebert, M.S., Neilson, J.R., and Sharp, P.A. (2007). MicroRNA sponges: competitive inhibitors of small RNAs in mammalian cells. Nat. Methods *4*, 721–726.
7. Gentner, B., Schira, G., Giustacchini, A., Amendola, M., Brown, B.D., Ponzoni, M., and Naldini, L. (2009). Stable knockdown of microRNA in vivo by lentiviral vectors. Nat. Methods *6*, 63–66.
8. Haraguchi, T., Ozaki, Y., and Iba, H. (2009). Vectors expressing efficient RNA decoys achieve the long-term suppression of specific microRNA activity in mammalian cells. Nucleic Acids Res. *37*, e43.
9. Vermeulen, A., Robertson, B., Dalby, A.B., Marshall, W.S., Karpilow, J., Leake, D., Khvorova, A., and Baskerville, S. (2007). Double-stranded regions are essential design components of potent inhibitors of RISC function. RNA *13*, 723–730.
10. Wang, X., Han, L., Zhang, A., Wang, G., Jia, Z., Yang, Y., Yue, X., Pu, P., Shen, C., and Kang, C. (2011). Adenovirus-mediated shRNAs for co-repression of miR-221 and miR-222 expression and function in glioblastoma cells. Oncol. Rep. *25*, 97–105.
11. Bak, R.O., Hollensen, A.K., Primo, M.N., Sørensen, C.D., and Mikkelsen, J.G. (2013). Potent microRNA suppression by RNA Pol II-transcribed 'Tough Decoy' inhibitors. RNA *19*, 280–293.
12. Sakurai, F., Furukawa, N., Higuchi, M., Okamoto, S., Ono, K., Yoshida, T., Kondoh, M., Yagi, K., Sakamoto, N., Katayama, K., and Mizuguchi, H. (2012). Suppression of hepatitis C virus replicon by adenovirus vector-mediated expression of tough decoy RNA against miR-122a. Virus Res. *165*, 214–218.
13. Xie, J., Ameres, S.L., Friedline, R., Hung, J.H., Zhang, Y., Xie, Q., Zhong, L., Su, Q., He, R., Li, M., et al. (2012). Long-term, efficient inhibition of microRNA function in mice using rAAV vectors. Nat. Methods *9*, 403–409.
14. Hollensen, A.K., Thomsen, R., Bak, R.O., Petersen, C.C., Ermegaard, E.R., Aagaard, L., Damgaard, C.K., and Mikkelsen, J.G. (2017). Improved microRNA suppression by WPRE-linked tough decoy microRNA sponges. RNA *23*, 1247–1258.
15. Hollensen, A.K., Bak, R.O., Haslund, D., and Mikkelsen, J.G. (2013). Suppression of microRNAs by dual-targeting and clustered Tough Decoy inhibitors. RNA Biol. *10*, 406–414.
16. Mockenhaupt, S., Grosse, S., Rupp, D., Bartenschlager, R., and Grimm, D. (2015). Alleviation of off-target effects from vector-encoded shRNAs via codelivered RNA decoys. Proc. Natl. Acad. Sci. USA *112*, E4007–E4016.
17. Michler, T., Große, S., Mockenhaupt, S., Röder, N., Stückler, F., Knapp, B., Ko, C., Heikenwalder, M., Protzer, U., and Grimm, D. (2016). Blocking sense-strand activity improves potency, safety and specificity of anti-hepatitis B virus short hairpin RNA. EMBO Mol. Med. *8*, 1082–1098.
18. Hansen, T.B., Jensen, T.I., Clausen, B.H., Bramsen, J.B., Finsen, B., Damgaard, C.K., and Kjems, J. (2013). Natural RNA circles function as efficient microRNA sponges. Nature *495*, 384–388.
19. Memczak, S., Jens, M., Elefsinioti, A., Torti, F., Krueger, J., Rybak, A., Maier, L., Mackowiak, S.D., Gregersen, L.H., Munschauer, M., et al. (2013). Circular RNAs are a large class of animal RNAs with regulatory potency. Nature *495*, 333–338.
20. Hansen, T.B., Wiklund, E.D., Bramsen, J.B., Villadsen, S.B., Statham, A.L., Clark, S.J., and Kjems, J. (2011). miRNA-dependent gene silencing involving Ago2-mediated cleavage of a circular antisense RNA. EMBO J. *30*, 4414–4422.
21. Hansen, T.B., Kjems, J., and Damgaard, C.K. (2013). Circular RNA and miR-7 in cancer. Cancer Res. *73*, 5609–5612.

22. Piwecka, M., Glažar, P., Hernandez-Miranda, L.R., Memczak, S., Wolf, S.A., Rybak-Wolf, A., Filipchyk, A., Klironomos, F., Cerda Jara, C.A., Fenske, P., et al. (2017). Loss of a mammalian circular RNA locus causes miRNA deregulation and affects brain function. Science *357*, eaam8526.

23. Geng, H.H., Li, R., Su, Y.M., Xiao, J., Pan, M., Cai, X.X., and Ji, X.P. (2016). The Circular RNA Cdr1as Promotes Myocardial Infarction by Mediating the Regulation of miR-7a on Its Target Genes Expression. PLoS ONE *11*, e0151753.

24. Xu, H., Guo, S., Li, W., and Yu, P. (2015). The circular RNA Cdr1as, via miR-7 and its targets, regulates insulin transcription and secretion in islet cells. Sci. Rep. *5*, 12453.

25. Sharma, N., Cai, Y., Bak, R.O., Jakobsen, M.R., Schrøder, L.D., and Mikkelsen, J.G. (2013). Efficient sleeping beauty DNA transposition from DNA minicircles. Mol. Ther. Nucleic Acids *2*, e74.

26. Kramer, M.C., Liang, D., Tatomer, D.C., Gold, B., March, Z.M., Cherry, S., and Wilusz, J.E. (2015). Combinatorial control of Drosophila circular RNA expression by intronic repeats, hnRNPs, and SR proteins. Genes Dev. *29*, 2168–2182.

27. Staunstrup, N.H., Madsen, J., Primo, M.N., Li, J., Liu, Y., Kragh, P.M., Li, R., Schmidt, M., Purup, S., Dagnæs-Hansen, F., et al. (2012). Development of transgenic cloned pig models of skin inflammation by DNA transposon-directed ectopic expression of human β1 and α2 integrin. PLoS ONE *7*, e36658.

OMTN, Volume *13*

Supplemental Information

Enhanced Tailored MicroRNA Sponge Activity of RNA Pol II-Transcribed TuD Hairpins Relative to Ectopically Expressed ciRS7-Derived circRNAs

Anne Kruse Hollensen, Sofie Andersen, Karina Hjorth, Rasmus O. Bak, Thomas B. Hansen, Jørgen Kjems, Lars Aagaard, Christian Kroun Damgaard, and Jacob Giehm Mikkelsen

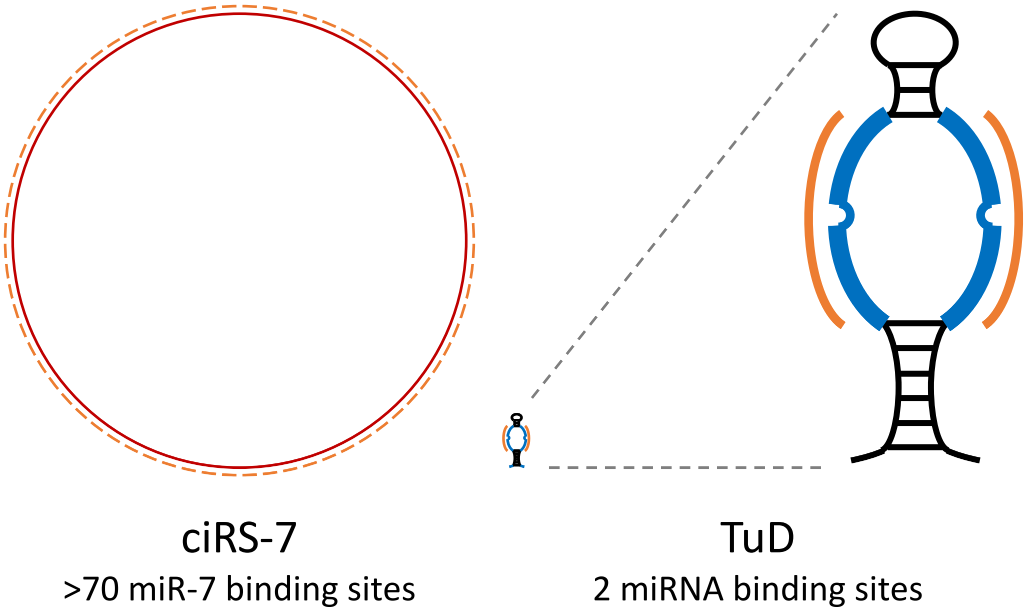

Supplementary figure S1. Schematic representation of ciRS-7 and a TuD hairpin. Processed miR-7 molecules binding to the sponges are shown in orange.

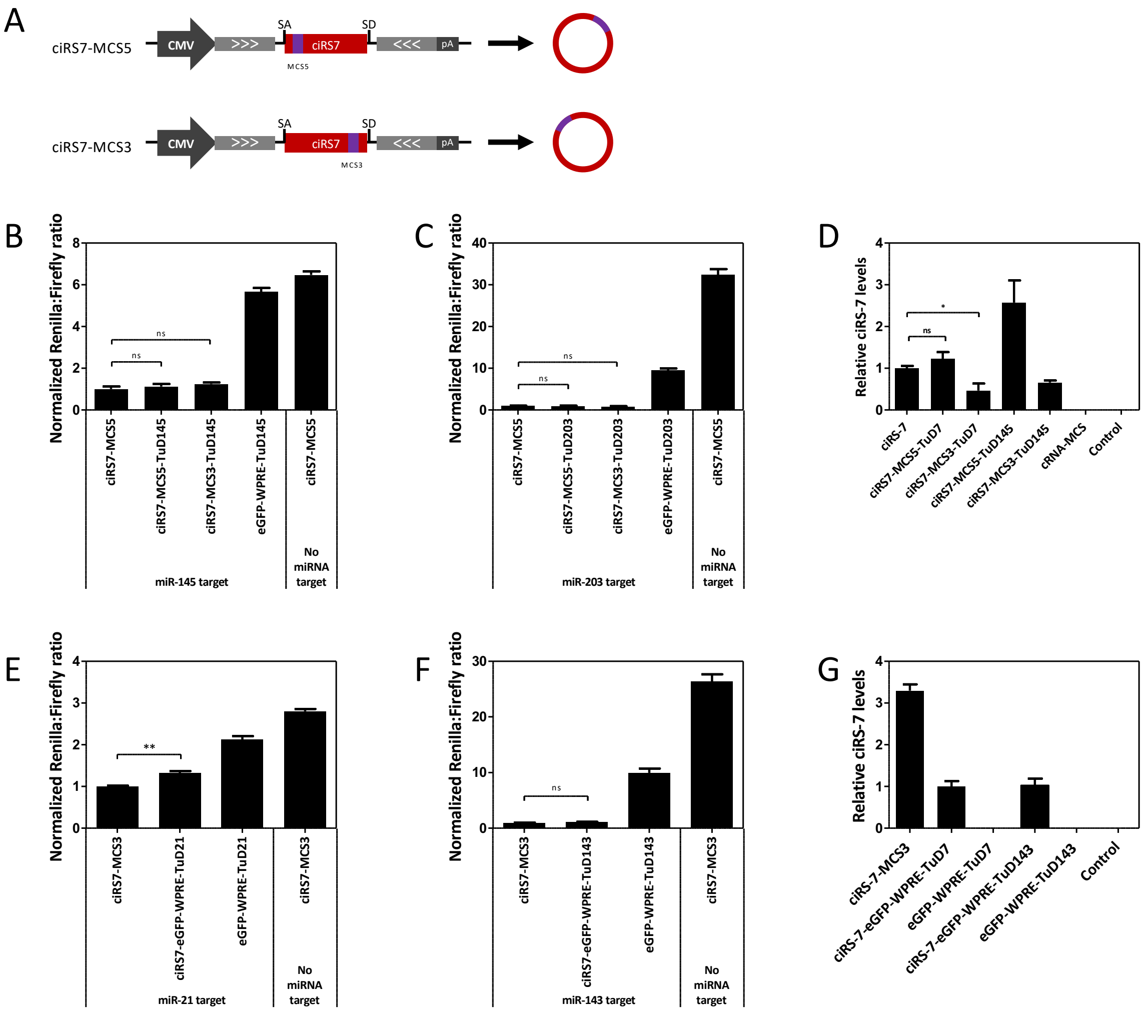

Supplementary figure S2. (A) Schematic representation of ciRS-7 expression plasmids containing multiple cloning sites in either the 5'- (MCS5) or 3'-end (MCS3) of ciRS-7. Dual-Glo luciferase assays evaluating miRNA suppression mediated by ciRS-7 carrying either TuDs targeting miR-145 (B) and -203 (C) or the eGFP-WPRE-TuD expression cassette encoding TuDs targeting miR-21 (E) and -143 (F). (D) and (G) Verification of circle formation of ciRS-7 and ciRS-7 carrying TuDs by TaqMan qPCR using primers and probe spanning the splice junction. (*) P < 0.05, (**) P < 0.01, (ns) not significant.

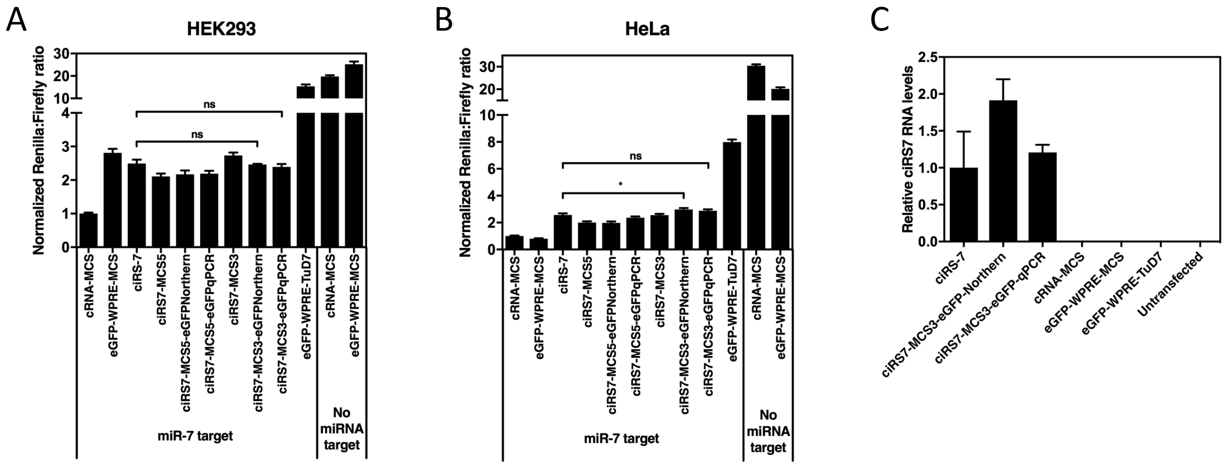

Supplementary figure S3. Dual-Glo luciferase assay in HEK293 (A) and HeLa (B) evaluating the miR-7 suppression mediated by ciRS-7 and ciRS-7 with primer and probe binding sites for either eGFP specific TaqMan qPCR (eGFP-qPCR) or Northern blot (eGFP-Northern) inserted in either ciRS7-MCS5 or ciRS7-MCS3. Both cell lines were transfected with equal molar amounts of plasmids. (C) ciRS-7 specific TaqMan qPCR using primers and probe spanning the splice junction. The same RNA samples were also used for the eGFP specific TaqMan qPCR shown in figure 3B and Northern blot shown in figure 3C. (*) $P < 0.05$, (ns) not significant.

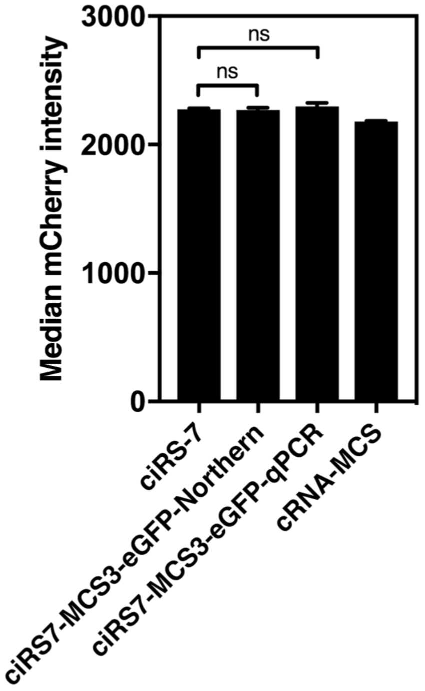

Supplementary figure S4. miR-7 suppression potential evaluated in HEK Flp-In T-rex miR7-4xmiR7-target cells with transiently expressed ciRS-7, ciRS-7-MCS3-eGFP-Northern, or ciRS-7-MCS3-eGFP-qPCR. The transfections were made using equal molar amounts of plasmids. (ns) not significant.

SUPPLEMENTARY TABLE S1: Primers

**pcDNA3/ciRS7-MCS5**	
**5141**	5’ - ccg ggg tac caa act cga gaa agg cgc gcc t - 3’
**5142**	5’ - ccg gag gcg cgc ctt tct cga gtt tgg tac c - 3’
**pcDNA3/ciRS7-MCS3**	
**5143**	5’ - ggt acc aaa ctc gag aaa ggc gcg ccg tct tcc atc aac tgg ctc aca a - 3’
**5144**	5’ - tga gcc agt tga tgg aag acg gcg cgc ctt tct cga gtt tgg tac ctt g - 3’
**pcDNA3/ciRS7-MCS5-TuD and pcDNA3/ciRS7-MCS3-TuD**	
**282**	5’ - aaa agg tac cgt atg aga cca ccc tag ccc tag ccc - 3’
**283**	5’ - aaa agg tac cca gag aga ccc agt aca agc - 3’
**pcDNA3/ciRS7-MCS5-WPRE and pcDNA3/ciRS7-MCS3-WPRE**	
**5175**	5’ - aaa ggt acc taa tca acc tct gga tta ca - 3’
**5176**	5’ - aaa ggc gcg ccg cgg gga ggc ggc cca aag g - 3’
**pcDNA3/ciRS7-eGFP-WPRE-TuD**	
**5137**	5’ - aaa gcg gcc gct ccg gaa tgg tga gca agg gcg agg ag - 3’
**5154**	5’ - aaa ggc gcg cct taa tta agc ggc cgc acc g - 3’
**pcDNA3/ciRS7-MCS5-eGFP-qPCR and pcDNA3/ciRS7-MCS3-eGFP-qPCR**	
**5244**	5’ - aaa ggt acc ctg ctg ccc gac aac cac - 3’
**5245**	5’ - aaa ggc gcg cct gtg atc gcg ctt ctc gtt - 3’
**pcDNA3/ciRS7-MCS5-eGFP-Northern and pcDNA3/ciRS7-MCS3-eGFP-Northern**	
**5230**	5’ - aaa ggt acc cta cgg cgt gca gtg ctt cag - 3’
**5231**	5’ - aaa ggc gcg cct tga aga agt cgt gct gct tc - 3’
**pcDNA3/CMV-eGFP-WPRE-TuD and pcDNA3/CMV-eGFP-WPRE-MCS**	
**5228**	5’ - aaa aag ctt gcc acc atg gtg agc aag ggc gag gag c - 3’
**5229**	5’ - aaa ggg ccc tta agc ggc cgc acc ggt ac - 3’

SUPPLEMENTARY TABLE S2: Primers and probes for TaqMan qPCR

**ciRS-7**	
**Forward primer**	5’ - gtc ttc cat caa ctg gct ca - 3’
**Reverse primer**	5’ - aac ttg aca cag gtg cca tc - 3’
**Probe**	5’ - Fam - ctt cca acg tct cca gtg tgc tga - BHQ1 - 3’
**eGFP**	
**Forward primer**	5’ - ctg ctg ccc gac aac cac - 3’
**Reverse primer**	5’ - tgt gat cgc gct tct cgt t - 3’
**Probe**	5’ - Fam - acc tga gca ccc agt ccg cc t - Tamra - 3’
**WPRE**	
**Forward primer**	5’ – ggc act gac aat tcc gtg gt - 3’
**Reverse primer**	5’ – agg gac gta gca gaa gga cg - 3’
**Probe**	5’ - Fam – acg tcc ttt cca tgg ctg ctc gc - BHQ1 - 3’
**RPLP0**	
**Forward primer**	5’ - ggc gac ctg gaa gtc caa ct - 3’
**Reverse primer**	5’ - cca tca gca cca cag cct tc - 3’
**Probe**	5’ - Fam - atc tgc tgc atc tgc ttg gag ccc a - Tamra - 3’

SUPPLEMENTARY TABLE S3: Probes for Northern blot

**eGFP**	5’ - ttg aag aag tcg tgc tgc ttc atg tgg tcg ggg tag cgg ctg aag cac tgc acg ccg tag - 3’
**B-actin**	5’ - gcc aga ttt tct cca tgt cgt ccc agt tgg tga cga tgc cgt gct cga tg - 3’
**ciRS-7**	5’ - ttg gaa gac ttg aag tcg ctg gaa gac ccg gag ttg ttg gaa gac ctt gac aca ggt gcc - 3’
**rps5**	5’ - gca gac agg ttt att ggg cag cag ctg gga aaa tca gcg gtt gga ctt ggc cac acg ctc - 3’
